# Enhanced microbiota-derived mucinases in colorectal cancer patients revealed by gut metagenome probing coupled with functional validation

**DOI:** 10.1128/aem.01903-25

**Published:** 2026-04-06

**Authors:** Yuming Li, Han Zhang, Baoyu Xiang, Yan Zhang, Menghui Zhang

**Affiliations:** 1 State Key Laboratory of Microbial Metabolism, Joint International Research Laboratory of Metabolic & Developmental Sciences, School of Life Sciences and Biotechnology, Shanghai Jiao Tong University 12474, Shanghai, China; 2 Key Laboratory of Systems Biomedicine (Ministry of Education), Shanghai Center for Systems Biomedicine, Center for Chemical Glycobiology, Zhangjiang Institute for Advanced Study, Shanghai Jiao Tong University, Shanghai, China; University of Illinois Urbana-Champaign, Urbana, Illinois, USA

**Keywords:** mucinase, gut metagenome, hidden Markov model, colorectal cancer

## Abstract

Mucinases produced by the gut microbiota play a dual role in regulating the integrity and renewal of the mucus layer, which is essential for maintaining gut homeostasis and human health. In this study, we constructed protein hidden Markov models based on 11 known mucinases and used them to systematically identify mucinase sequences from gut metagenome-assembled genomes derived from 80 colorectal cancer (CRC) patients and 86 healthy (Healthy) subjects. A total of 1,869 mucinases were detected, widely distributed across the studied cohorts, with the majority originating from *Bacteroides*, *Phocaeicola*, and *Akkermansia* species. Further analysis identified 42 mucinases that differed significantly in abundance between the two groups, all of which were enriched in CRC patients. Taxonomic attribution revealed that, in CRC patients, these mucinases were primarily derived from *Bacteroides* (36.0%), *Phocaeicola* (30.6%), *Akkermansia* (8.8%), *Alistipes* (8.6%), and *Escherichia* (6.4%), whereas in Healthy subjects, they mainly originated from *Bacteroides* (26.1%), *Akkermansia* (22.7%), and *Phocaeicola* (20.3%), with a notably higher proportion from *Akkermansia*. Among the 42 mucinases, WL42 and LLN1 exhibited significantly higher abundance levels compared to the others. Phylogenetic and predicted structural analyses suggested that these two mucinases belonged to the M60 and M98 families, respectively. Functional validation through co-incubation experiments demonstrated that both mucinases could cleave the glycosylated MUC1 and MUC2 substrates, but not the corresponding non-glycosylated proteins. These findings confirm the feasibility of discovering novel mucinases directly from gut metagenomic data and provide insights into their potential roles in health and disease.

**IMPORTANCE:**

Our study established a feasible bioinformatics pipeline for the systematic identification of microbial mucinases within the gut microbiome, providing a methodological foundation for large-scale mining of functionally active mucin-degrading enzymes. We identified 42 mucinases significantly enriched in CRC patients, suggesting their potential involvement in CRC pathogenesis. Among them, two mucinases were experimentally validated for their ability to degrade mucin, offering direct functional evidence of their capacity to disrupt the mucosal barrier. Genus-level metagenomic profiling further identified *Bacteroides*, *Phocaeicola*, and *Akkermansia* as major mucinase-producing genera. Maintaining the secretory balance of these mucinase-producing bacteria might be crucial for ameliorating intestinal barrier dysfunction in CRC patients. The findings of this study offer critical insights into the microbial origins and potential mechanistic contributions of mucinases in colorectal cancer, underscoring their relevance in mucus barrier breakdown and disease progression.

## INTRODUCTION

The intestinal barrier is a critical defense system maintaining human health (1, 2). Increasing evidence indicates that the disruption of the intestinal mucosal barrier can lead to a range of consequences, including microbial and toxin invasion, ultimately contributing to disease progression (3). The intestinal mucus layer, a key component of this barrier, plays a pivotal role in maintaining intestinal homeostasis and overall host health (4, 5). As the physical barrier closest to the intestinal lumen, the mucus layer not only prevents pathogens and toxins from accessing epithelial cells but also provides a suitable microenvironment and nutrients for symbiotic microorganisms, thereby playing a significant role in host-microbe interaction (6).

The intestinal mucus layer is primarily composed of various mucins, which are high-molecular-weight O-glycoproteins (7, 8). These mucins are essential for providing physical protection and regulating the distribution of water, ions, and immune mediators within the intestine (9). Furthermore, as a site of microbial colonization and a nutritional niche, the mucus forms a gel-like network at the interface between the intestinal lumen and the epithelial cells, contributing to the maintenance of gut microbiota homeostasis and digestive system stability. In the colon and rectum, the secreted mucus layer is dominated by MUC2, a goblet cell-derived secreted mucin that forms the inner and outer mucus layers (10). In addition, MUC1 is a transmembrane mucin localized to the apical surface of epithelial cells beneath the mucus layer, and its aberrant abundance and glycosylation are frequently observed across multiple cancers (11, 12). The quality and abundance of these two mucins are vital for the integrity of the colonic mucus layer.

Mucinases are proteases derived from the gut microbiota with the ability to specifically recognize and cleave glycoproteins in a glycan-dependent manner, thereby potentially influencing the intestinal mucus barrier (13, 14). These enzymes play a dual role: they help maintain mucus homeostasis by regulating its turnover, yet excessive activity can compromise barrier integrity, promote inflammation, and facilitate pathogen invasion (15, 16). To date, 11 types of mucinases have been identified, but their individual contributions to gut barrier dysfunction remain unclear (13).

Colorectal cancer (CRC) provides a valuable context for mucinase research, characterized by profound intestinal barrier disruption and dramatic shifts in the gut microbial community (17–19). Mucinases may critically influence host-microbe interactions and directly contribute to carcinogenesis through barrier compromise. Notably, CRC patients exhibit marked alterations in glycoprotein profiles and mucin composition, resulting in impaired function of the intestinal glycan barrier—a crucial defensive mechanism (1, 9). These interconnected features make CRC a valuable model for investigating mucinase activity and its pathological implications in cancer progression.

**Fig 1:**
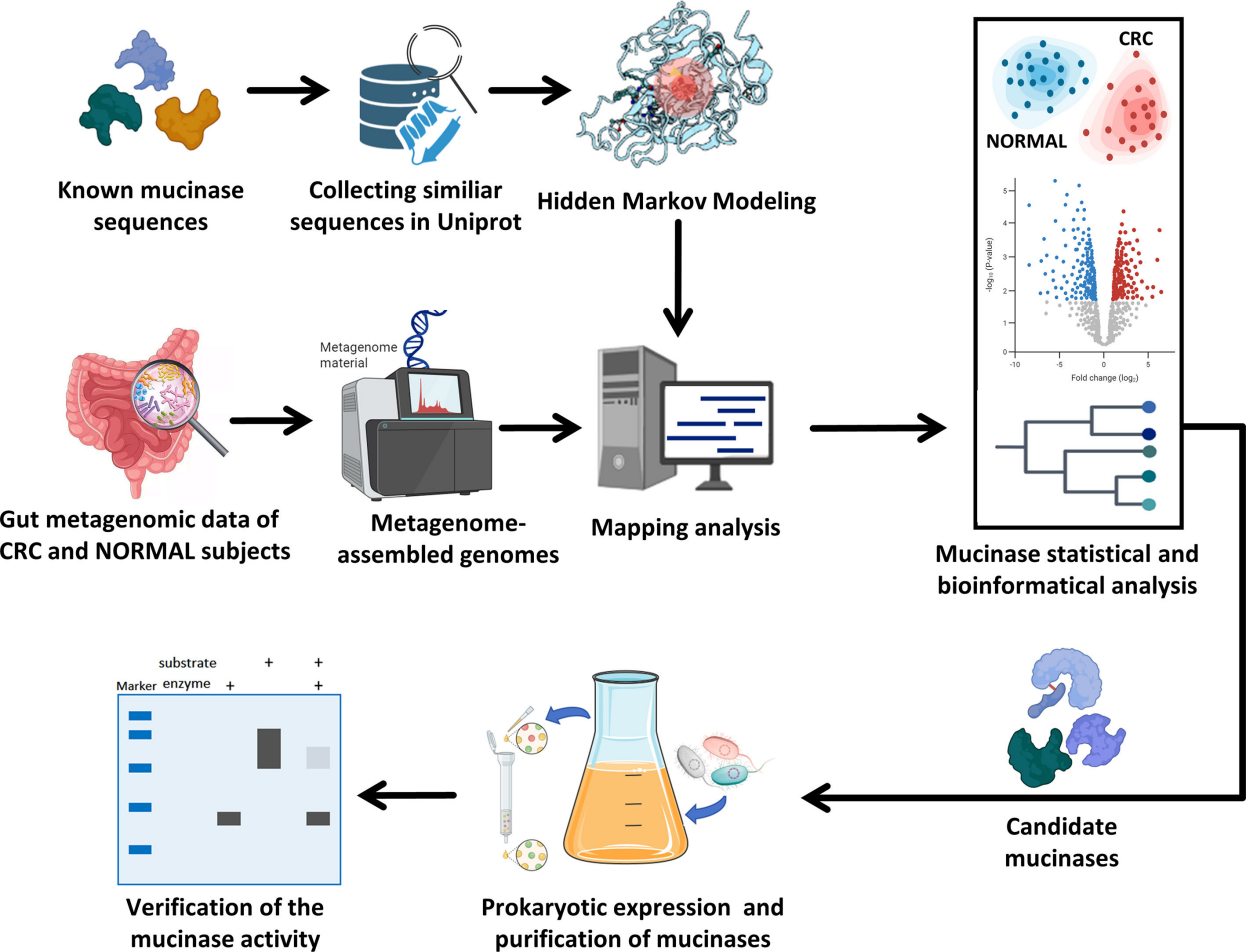
Mucinase mining schematic. HMMs built from known mucinases were used to screen gut metagenomes from CRC patients and Healthy controls. Hits were quantified in MAGs, followed by statistical/bioinformatic analyses to select mucinase candidates for activity validation. Elements of the schematic were created with BioRender (https://www.biorender.com/).

In this study, we developed a mucinase-mining pipeline integrating bioinformatic screening with functional validation (Fig. 1). Starting from the 11 known mucinases, we first collected a larger protein data set from the UniProt database based on predefined sequence and structural similarity and constructed hidden Markov models (HMMs) to extract features of these proteins. These HMMs were then used to match metagenome-assembled genomes (MAGs) from CRC patients and Healthy subjects to identify putative mucinases. Candidate mucinases were further prioritized using statistical tests for differential abundance between the two groups, followed by predicted structural comparisons and phylogenetic analyses. We also analyzed the bacterial sources of the identified mucinases. Finally, selected candidates were functionally validated through prokaryotic expression, purification, and co-incubation assays.

## RESULTS

### Overall mucinases detected from gut metagenomic data of CRC patients and Healthy subjects

The 11 mucinases (13) (BT4244, Amuc0627, Amuc0908, Amuc1514, ZmpB, ZmpC, IMPa, CpaA, Pic, OgpA, and StcE) used for HMM modeling were classified into 8 families in MEROPS (the peptidase database, https://www.ebi.ac.uk/merops/) (Fig. S1a). At the start of this study, these represented the set of well-characterized mucinases. We retrieved a total of 3,061 protein sequences from UniProt with sequence similarity greater than 50% to the 11 mucinases. This number was expanded to 102,630 after BLAST analysis against the bacterial whole-genome database. After quality control (protein length between 300 and 3,000 amino acids), 22,263 sequences were retained for HMM construction. Of these, most were present at low abundance and detected in only a few subjects. Applying thresholds of total abundance > 16 and subject coverage > 5, only 1,869 HMMs were retained for further analysis.

### Differential mucinases in CRC patients and Healthy subjects

**Fig 2:**
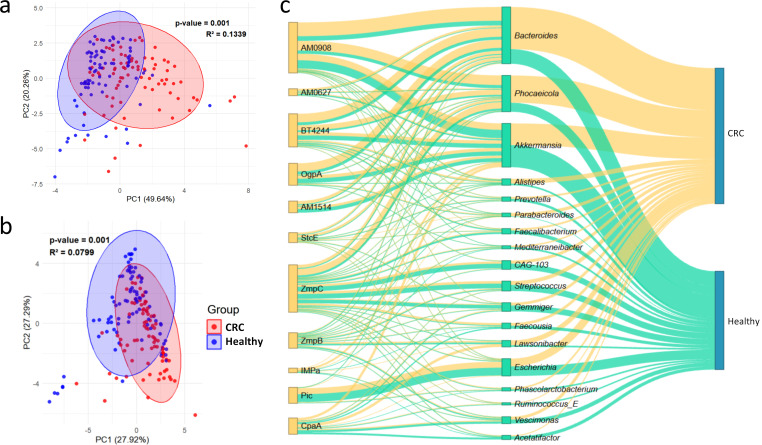
Unsupervised compositional analyses of mucinases and bacteria in CRC and Healthy subjects. (**a and b**) PCA based on (**a**) 1,869 mucinase HMMs and (**b**) the bacterial composition. The elliptic confidence is set to 95%, and the *P*-value and *R*^2^ are calculated using the PERMANOVA method. (**c**) Sankey diagram summarizing the 1,869 mucinase HMMs by their assignment to the 11 seed mucinase categories, inferred producing genera, and host status (CRC vs Healthy). Link widths indicate aggregated abundance (or inferred contribution), and colors denote cohort association (CRC, yellow; Healthy, cyan).

Principal component analysis (PCA) of the 1,869 detected mucinase HMMs revealed significant separation between the CRC patients and the Healthy subjects (Fig. 2a), consistent with the separation observed for the bacterial community (Fig. 2b). The Procrustes analysis showed a strong correlation between the microbiota and mucinase matrices (*r* = 0.623, *P* = 0.001), indicating a significant association between intestinal microbiota and detected mucinases.

Based on the mapping results of 1,869 mucinases, we investigated the source bacteria of the 11 mucinases and their distribution across the two groups (Fig. 2c). *Bacteroides*, *Phocaeicola*, *Akkermansia*, and *Escherichia* were the four most abundant bacterial genera. Additionally, mucinase contributors *Alistipes*, *Parabacteroides*, and *Lawsonibacter* were predominantly found in CRC patients, while *Streptococcus*, *Acetatifactor,* and *Gemmiger* were more prevalent in Healthy individuals. Among the detected mucinases, Amuc0908 (AM0908), ZmpC, and BT4244 were the most abundant. AM0908 and BT4244 tended to accumulate in CRC patients, whereas CpaA, ZmpB, and ZmpC were more common in Healthy individuals. All results from unsupervised analyses indicated that the distribution and bacterial sources of mucinases in CRC patients differ significantly from those in Healthy individuals.

**Fig 3:**
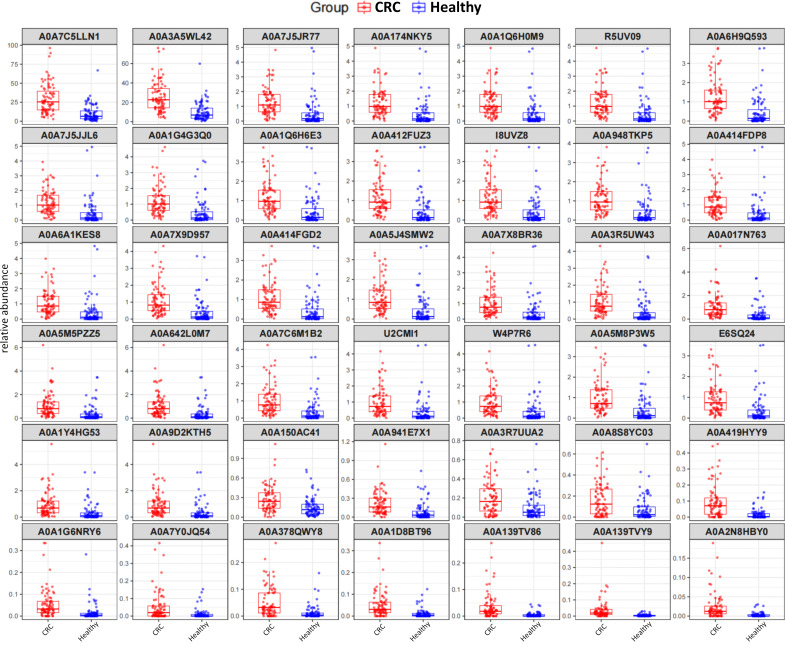
The abundances of 42 mucinases between the CRC and the Healthy. After removing samples with extremely low sequencing read counts in both groups, CRC (*n* = 79) and Healthy (*n* = 85) are shown in red and blue, respectively. Mucinases are ordered by descending abundance across the cohort.

Partial least squares discriminant analysis (PLS-DA) with 10-fold cross-validation revealed that using the 1,869 HMMs, a PLS model with five components achieved 78.5% accuracy in distinguishing CRC from Healthy subjects. This model identified 105 HMMs as significant distinguishers at *P* = 0.05, and 42 of them exhibited highly significant differential abundance between the two groups (unpaired *t*-test, FDR-adjusted *P* < 0.0001). Notably, all 42 proteins were enriched in the CRC population (Fig. 3). To enhance disease relevance, we trained three machine learning models (Logistic Regression, Random Forest, and Support Vector Machine) using the abundance matrix of these 42 mucinases (Fig. S3a). The RF model showed the best performance, with an area under the curve of 0.917 (Fig. S3b).

**Fig 4:**
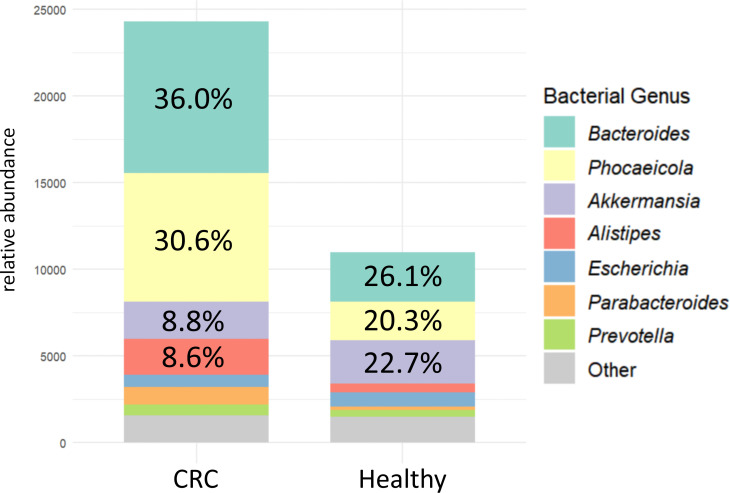
Abundance distribution of source bacteria of the 42 mucinases in CRC patients and Healthy subjects. The vertical axis measures the normalized abundance of the bacterial strains. The proportion of each strain in the corresponding group is indicated on the corresponding bar chart.

The intestinal mucinase-producing bacteria differ between CRC patients and Healthy individuals (Fig. 4). These 42 mucinases exhibited not only higher total abundance in CRC but also distinct bacterial sources. In the intestinal microenvironment of CRC patients, mucinase genes were primarily predicted to derive from the genera *Bacteroides* (36.0%) and *Phocaeicola* (30.6%), with significant contributions from *Akkermansia* (8.8%), *Alistipes* (8.6%), *Escherichia* (6.4%), *Prevotella*, and *Parabacteroides*. In comparison, the mucinase-producing bacteria in Healthy individuals showed a different composition, with *Bacteroides* (26.1%), *Phocaeicola* (20.3%), and *Akkermansia* (22.7%) as the major predicted producers. Other genera, including *Alistipes*, *Escherichia*, *Prevotella*, and *Parabacteroides*, were also predicted as participants in mucin degradation in this control group.

### Two distinct mucinases stand out

**Fig 5:**
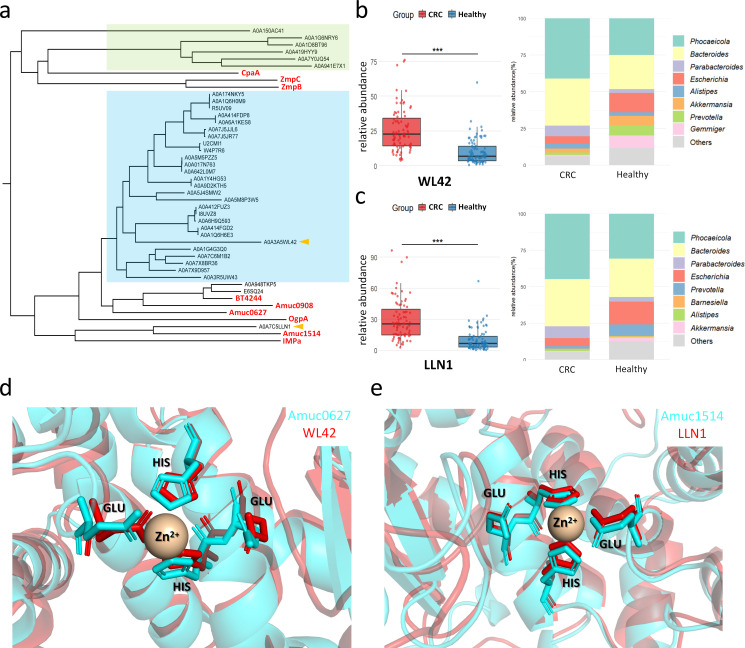
Abundance distribution and bacterial source track of WL42 and LLN1 mucinases in CRC and Healthy subjects. (**a**) Phylogenetic tree of 42 candidate mucinases with the 11 reference mucinases. The reference mucinases are marked in red, and the two selected candidates (WL42 and LLN1) are indicated by the orange triangles. Blue and green shading denotes the two candidate clusters. (**b and c**) Abundance of WL42 (**b**) and LLN1 (**c**) in CRC and Healthy subjects and the relative contributions of inferred microbial producers. ***, *P* < 0.001. (**d and e**) Structure alignment of (**d**) Amuc0627 and WL42 and (**e**) Amuc1514 and LLN1. The reference structures (Amuc0627/Amuc1514) are colored in cyan, and the predicted structures of candidates (WL42/LLN1) are colored in red.

By screening mucinase abundance in the intestinal tract, we identified A0A3A5WL42 and A0A7C5LLN1 (hereafter referred to as WL42 and LLN1) as outliers among the 42 most abundant mucinases (Fig. 3). Among the remaining 40 mucinases, phylogenetic analysis (Fig. 5a; Fig. S4) revealed that 26 (65%) were more closely related to Amuc0627 and 6 (15%) to CpaA, indicating that most candidates are evolutionarily related to known mucinases.

Based on MAG-HMM comparisons, we analyzed the abundance of WL42 and LLN1 in CRC and Healthy groups and performed bacterial source tracking (Fig. 5b and c). In the CRC group, *Bacteroides*, *Phocaeicola*, and *Parabacteroides* were the main contributors to WL42 and LLN1, with lower inferred contributions in the Healthy group. In contrast, in the Healthy group, *Gemmiger*, *Akkermansia*, and *Prevotella* showed higher contribution to WL42, while *Escherichia* and *Prevotella* showed higher contribution to LLN1. The phylogenetic tree (Fig. 5a) indicated that the sequence of WL42 was similar to that of Amuc0627 among all known mucinases (evolutionary distance ~2.77), while the sequence of LLN1 was more similar to that of Amuc1514 (evolutionary distance ~2.24), suggesting that the former has a closer relationship with the M60 family and the latter has a closer relationship with the M98 family.

To increase confidence in the predicted mucinases, we aligned key regions (including Zn^2+^-binding sites and catalytic residues) between WL42 and Amuc0627, and between LLN1 and Amuc1514, respectively (Fig. S2a and b). Sequence analysis showed that the active-site and metal-binding residues are highly conserved across WL42, LLN1, Amuc0627, and Amuc1514. WL42 and Amuc0627, as well as LLN1 and Amuc1514, are highly similar in sequence, especially in the four residues surrounding the Zn^2+^-binding site. We next used AlphaFold3 to predict the structure of WL42 and compared it with Amuc0627. Structure alignment indicated that WL42 contains a conserved HEXXH motif (residues 774–778), which corresponds to the Zn^2+^-binding site in Amuc0627 and is essential for its catalytic activity. By analogy, we propose that the corresponding motif in WL42 likely serves a similar catalytic function (Fig. 5d). Comparable results were observed with LLN1 relative to Amuc1514 (Fig. 5e). These results suggested that WL42 and LLN1 share many characteristics of mucinases.

### Functional validation of the two identified mucinases

**Fig 6:**
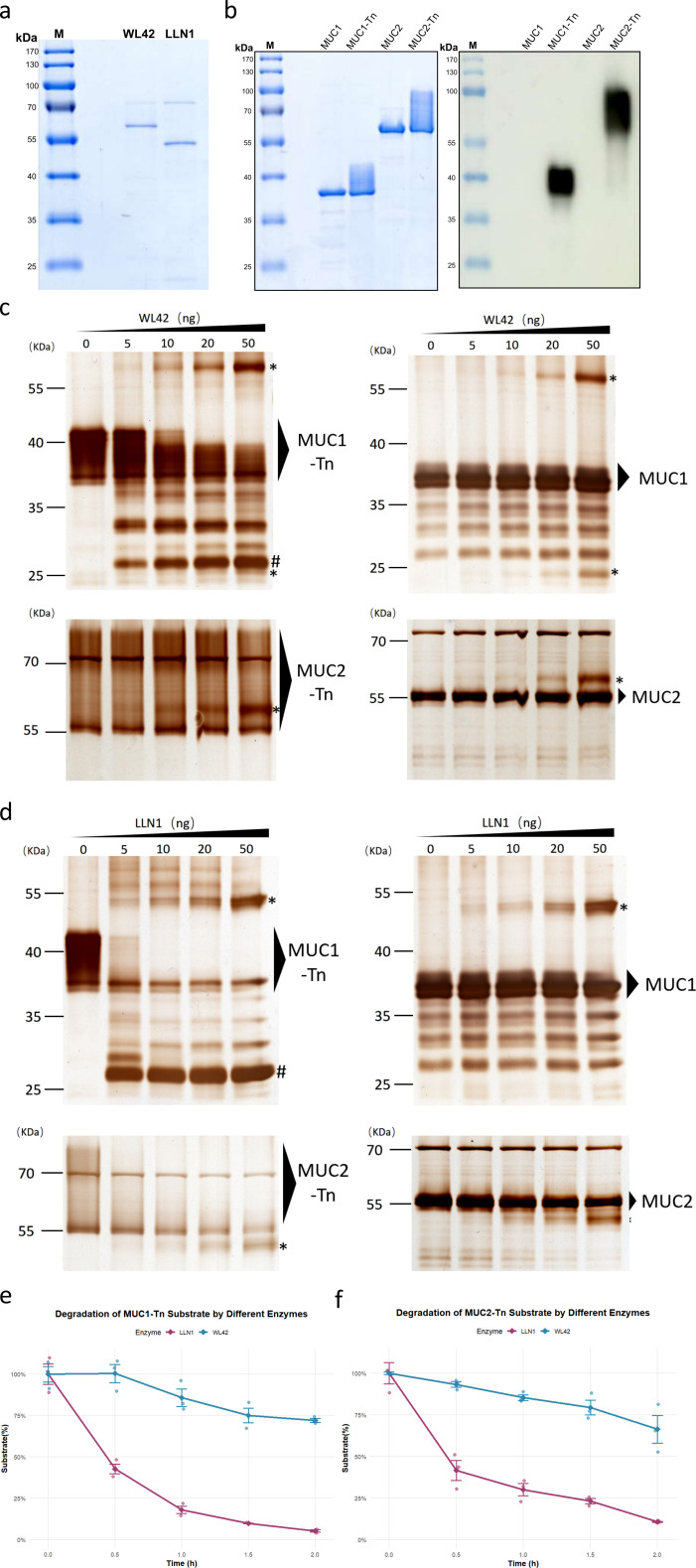
Purification and functional validation of WL42 and LLN1. (**a**) SDS-PAGE analysis of purified recombinant WL42 and LLN1. (**b**) Coomassie-stained SDS-PAGE (left) and *Vicia villosa* agglutinin lectin blot (right) of four protein substrates: non-glycosylated MUC1 and MUC2, and their O-glycosylated forms, MUC1-Tn and MUC2-Tn. (**c and d**) Silver-stained SDS-PAGE analysis of (**c**) WL42 and (**d**) LLN1 (dose titration) digestion of four protein substrates. Mucinases (0–50 ng) and substrates (500 ng) were incubated for 8 h at 37°C. Mucinase bands are marked with an asterisk (*), and the digestion product bands are indicated by a hashtag (#). (**e and f**) Time-course quantification of (**e**) MUC1-Tn and (**f**) MUC2-Tn degradation by WL42 and LLN1 using densitometry (grayscale analysis). Mucinases (50 ng) and substrates (500 ng) were incubated for 0–2 h at 37°C.

To assess the proteolytic activity of WL42 and LLN1, recombinant versions of both proteins were produced and purified. Following SDS-PAGE analysis, recombinant WL42 and LLN1 migrated at approximately 66 and 54 kDa, respectively (Fig. 6a). We tested four mucin proteins as substrates: O-GalNAc-glycosylated MUC1 and MUC2 (denoted as MUC1-Tn and MUC2-Tn), and their non-glycosylated counterparts (MUC1 and MUC2, see Fig. 6b). Enzymatic cleavage assays showed that WL42 and LLN1 both cleaved the Tn-glycosylated mucin substrates. Specifically, WL42 cleaved glycosylated MUC1 but showed only weak activity toward glycosylated MUC2 (Fig. 6c), whereas LLN1 efficiently cleaved both glycosylated forms (Fig. 6d). Notably, neither enzyme could cleave the protein substrate lacking glycosylation. Consistent with these endpoint assays, time-course experiments (0–2 h) monitoring depletion of the Tn-glycosylated substrates further confirmed glycan-dependent proteolysis by both enzymes, with LLN1 displaying consistently higher activity on both MUC1-Tn and MUC2-Tn (Fig. 6e and f). Taken together, *in vitro* enzymatic assays demonstrate that WL42 and LLN1 cleave mucins in a glycan-dependent manner, with LLN1 exhibiting stronger cleavage activity on both glycosylated substrates.

## DISCUSSION

The human mucus layer contains highly O-glycosylated proteins, forming the frontline of the intestinal barrier that interacts with gut microbes (5, 15, 16). In this study, we developed a pipeline to identify microbial-derived mucinases capable of glycan-dependent cleavage of mucin substrates using a public metagenomic data set from 80 CRC patients and 86 Healthy subjects. We detected over 20,000 potential mucinases in the metagenome, but only 1,869 were widely distributed across subjects. Among the 42 mucinases most significantly differentiating CRC from Healthy subjects, all were enriched abundance in the CRC group (Fig. 3). Functional validation of two of these mucinases, WL42 and LLN1, confirmed their ability to cleave glycosylated MUC1 and MUC2 substrates (Fig. 6e and f). The gut microbiota is a rich reservoir of natural enzymes, and our findings demonstrate the feasibility of discovering novel mucinases from gut metagenomes.

The 42 most significantly differentiated mucinases were all upregulated in the CRC group (Fig. 3), showing strong predictive power for CRC (Fig. S3a and b). In our previous clinical studies, we observed decreased butyrate-producing bacteria (20) and increased levels of gut flora metabolites (21) in the serum of CRC patients, suggesting intestinal barrier dysfunction. The discovery of mucinase upregulation in this study provides a specific pathway for intestinal barrier damage and highlights the critical role of gut bacteria-derived mucinases in CRC progression.

After stepwise data mining, we selected WL42 and LLN1 for functional validation, primarily due to their relatively higher and differential abundance. WL42 was predicted to belong to the M60 family, and LLN1 to the M98 family in the MEROPS database (Fig. 5c). Previous studies have shown that M60-family mucinases can degrade mucin layers (22), and that the M98 family mucinases tend to cleave mucins with truncated or sparse O-glycans (22, 23), suggesting that both families are closely related to intestinal mucin breakdown. The successful functional validation of WL42 and LLN1 supported the effectiveness of our candidate selection strategy. Our mucinase-mining pipeline provides a general framework for future discovery of novel enzymes. Moreover, since the presence of truncated glycoproteins and mucus layer thinning are hallmarks of intestinal mucus barrier dysfunction (24), we deduced that increased abundance of M60- and M98-family mucinases in the gut may serve as indicators of intestinal barrier impairment in CRC.

Previous studies have extensively compared the microbiota of tumor tissues and intestinal mucosa in CRC patients (17, 19, 25) and reported increased relative abundance (RA) of *Akkermansia* and *Bacteroides* in the intestine of CRC patients, which has been linked to intestinal inflammation and barrier dysfunction (3). However, our mucinase-focused analysis indicates that functional alterations may matter more than taxonomic shifts, and that strain-level assignment of specific functions is critical for identifying the main contributors. In our investigation, both *Bacteroides* and *Akkermansia* were identified as major producers of intestinal mucinases in both CRC and Healthy subjects (Fig. 4). *Bacteroides* was enriched in the CRC and contributed more dominantly to the production of mucinases WL42 and LLN1. Notably, *Bacteroides* and *Phocaeicola* were the primary sources of differentially abundant mucinases in this study, suggesting a potential association with intestinal barrier function. By comparison, *Akkermansia* showed a distinct pattern: although it was enriched in the CRC, its contribution to WL42 and LLN1 was lower than in the Healthy group. Additionally, *Akkermansia* was not a major source of WL42 and LLN1, indicating a weaker link between these candidates and *Akkermansia*-associated mucin degradation in CRC. Previous studies have reported that *Akkermansia* may stimulate intestinal goblet cells to produce more mucin by degrading an appropriate amount of mucin (26, 27). Consistent with this model, our results suggest that *Akkermansia*-encoded mucinases might be beneficial, or at least less harmful, for intestinal barrier maintenance.

In this study, we used the GTDB database for MAG annotation to enable the tracing of mucinase-producing strains and identify candidates of potential therapeutic interest. While this genomic approach precisely links functional enzymes to their microbial hosts, the lack of culture-based validation remains a key limitation. Future isolation and experimental verification of these strains would validate their enzymatic potential and bridge the gap between genomic predictions and biotechnological applications.

Structure predictions by AlphaFold3 of WL42 and LLN1 proteins indicated that both proteins were structurally similar to the known mucinases (Fig. 5d and e), particularly in key catalytic residues. However, the two mucinases differ significantly in their ability to cleave mucin substrates. These findings suggest that functional prediction of proteases cannot rely solely on structural alignment and requires experimental validation. While recent advances in protein structure prediction offer valuable insights for optimizing enzyme systems (28), significant challenges remain. Although molecular docking methods have enabled the initial matching of mucoproteases to their glycoprotein substrates (29), the inherent flexibility of glycan chains and the uncertainty of the predicted protease structure remain challenges for accurate glycoprotein-mucoprotease docking studies. Furthermore, the precise molecular mechanisms underlying mucinase activity demand further investigation to fully understand their substrate specificity and catalytic behavior.

As this study is a pilot investigation aimed at assessing the feasibility of mining novel mucinases from metagenomes, we focused on a narrowed region of mucin-degradable enzymes and selected only two mucinases for functional validation. Given the limitations of the current research, future studies should investigate cooperative relationships among broader arrays of mucin-degradable enzymes and their associated mucin-secreting microbiota to better address gut barrier-related pathologies. Importantly, recent studies have offered new perspectives on potential therapeutic applications, suggesting that engineered mucinases could be developed as tumor-targeting tools, thereby inspiring future translational research in this field.

In conclusion, using our mucinase mining pipeline that integrates bioinformatic screening with functional validation, we identified novel microbial-derived mucinases in the human gut metagenome by comparing the CRC and Healthy groups. The two computationally screened mucinases, WL42 and LLN1, which were primarily attributed to *Bacteroides* and *Phocaeicola*, may serve as indicators of gut barrier impairment during the onset and progression of CRC. Our process of discovering mucinases contributes to understanding the underlying mechanisms of diseases involving gut barrier dysfunction and lays the foundation for disease diagnosis and treatment.

## MATERIALS AND METHODS

### HMM modeling

The HMM modeling was initialized with 11 well-characterized mucinases: BT4244, Amuc0627, Amuc0908, Amuc1514, ZmpB, ZmpC, IMPa, CpaA, Pic, OgpA, and StcE (13). First, a protein data set was expanded by searching UniProt for relevant protein names and collecting sequences with similarity >50% to these mucinases. The collections of proteins were re-enlarged by BLAST searching the proteins against the bacterial whole-genome data in the UniProt database (size 111.67 GB, July 2023) with a threshold of 1e-5. Sequences 300–3,000 amino acids in length were selected, clustered with CD-HIT software (30) at 80% sequence identity, and functionally annotated using HMMER software (31) against the Pfam database (size 1.52 GB, September 2023) with a 1e-10 threshold. Based on Pfam annotations, proteins containing protease-related domains were retained. Finally, HMMs were constructed using HMMER software with the aligned sequences of the remaining proteins, provided by MAFFT.

### Metagenomic data processing

The metagenomic data (accession number PRJNA731589) used in this study to mine mucinases were downloaded from the National Library of Medicine website. This data set contains 80 colorectal cancer patients (32 females and 48 males) and 86 Healthy control subjects (34 females and 52 males). The metagenomic data were preprocessed with fastp version 0.23.4 (32) to filter low-quality sequences and with bowtie2 version 2.5.1 (33) to remove human contamination using GRCh38.p14 with default parameters. MEGAHIT version 1.2.29 (34) was used to assemble high-quality reads to obtain contigs, and contigs less than 200 bp in length were removed. The high-quality reads were then matched to contigs using bowtie2 and samtools version 1.9 (35), and bins were obtained with MetaBAT2 version 2.12.1 (36). CheckM2 version 1.0.1 (37) was used to predict the quality of the bins, and dRep version 3.4.3 (38) was applied to cluster high-quality bins (integrity > 95% and pollution < 5%) at the species level with 95% sequence similarity threshold. Taxonomic assignment and annotation of the MAGs were performed with GTDB-TK version 2.3.2 (39) based on the GTDB database r214. The abundances of MAGs in each sample were calculated with CoverM version 0.6.1 (40). The mean method was applied, and the min-read-percent-identity and min-read-aligned-percent were both set to 90%, and the min-covered-fraction was set to 50%.

### Calculation of mucinase abundance levels in population

In this study, each predicted mucinase identified from the metagenomic data corresponds to an HMM in our previously constructed HMM model. The abundance of a mucinase in a sample was calculated by summing the number of HMM hits across MAGs and then normalizing by metagenomic sequencing depth and HMM profile length.

To quantitatively assess the functional potential of microbial communities, we implemented an abundance-normalized approach for interpreting HMM search results. This methodology is based on gene presence in metagenome-assembled genomes and microbial population dynamics.

The hmmsearch module in HMMER was used to search the predicted protein sequences from MAGs against our HMM profiles and quantify HMM-MAG matches. The threshold was set to 1e-5, and other parameters were set to default. The raw hits were recorded in integer form and used to construct an MAG-HMM count matrix.

The relative abundance of each HMM across different samples was calculated as follows.

(i) The relative abundance (RA) of the *j*th HMM in the *i*th MAG in a sample was calculated as


$$RA_{ij}=\frac{N_{ij}}{L_{j}\times G_{i}}$$

where *N_ij_* is the number of significant hits of the *j*th HMM in the *i*th MAG, *L_j_* is the length of the *j*th HMM model (kb), and *G_i_* is the length of the *i*th MAG (Mb).

Then, the relative contribution of the *i*th MAG to the *j*th HMM in a sample was calculated with *N_ij_* divided by the total number of significant hits of the *j*th HMM across all MAGs in that sample.

(ii) Normalizing HMM hit counts by MAG abundance. As described above, MAGs were processed with CoverM, resulting in a quantitative abundance matrix (*A_ik_*), where each entry represents the mean sequencing coverage depth of a specific MAG *i* in a given sample *k*. For each sample, the RA data were converted into sample-specific quantitative values by multiplying by the corresponding MAG abundance metrics. Following the methodology proposed by Li et al. (41), the normalized hit count for the *i*th MAG and *j*th HMM in sample *k* was computed as


$$H_{ijk}^{normalized}=A_{ik}\times RA_{ij}$$

(iii) The total representation of each HMM within each sample (*T*_*jk*_) was defined as the sum of all normalized MAG-specific hit counts:


$$T_{jk}=\sum_{i=1}^{N}H_{ijk}^{\text{normalized}}$$

Collectively, this normalization pipeline links genomic data to protein HMM profiles, generating standardized metrics that reflect the comparative abundance of mucin-degrading enzymes across all samples.

### Candidate mucinase filtering

To narrow down the pool of candidate mucinases, we focused on those with higher abundance and significant differential abundance between CRC patients and Healthy controls. This was achieved through multivariate statistical analysis, including PLS-DA. Then, MEGA (version 10.2.6) software (42) was used to construct a phylogenetic tree via multiple sequence alignment using the ClustalW method with default parameters, and the tree was drawn using the neighbor-joining method with a step size of 1,000 and other default parameters. This clustering facilitated the selection of representative candidate mucinases within each cluster. Finally, the structures of the targeted mucinases were predicted with AlphaFold3 (https://alphafoldserver.com) (28). The protein structure comparison and mapping were performed with PyMOL (version 2.3) (43).

### Expression and purification of mucinases and substrate proteins

The coding sequences of each target mucinases were synthesized and cloned into the pET28a expression vector (GENERAL BIOL, China). The plasmids were transformed into *Escherichia coli* BL21 (DE3), and transformants were cultured in LB medium supplemented with kanamycin (50 μg/mL) at 37°C until OD_600_ reached 0.6–0.8. Protein expression was induced by the addition of 0.4 mM IPTG at 16°C for 16 h. The cells were harvested by centrifugation at 4°C and 5,000 *g* for 10 min and resuspended in binding buffer (25 mM Tris-HCl, 150 mM NaCl, 10 mM imidazole, and 1 mM PMSF; pH 8.0). After disruption by high pressure at 4°C, the supernatant was collected by centrifugation at 4°C and 12,000 *g* for 20 min. His-tagged mucinases were purified using Ni-IDA Beads 6FF (Smart-Lifesciences, China).

The expression and purification of the substrate proteins (MUC1 and MUC2) were performed as described above. Briefly, recombinant MUC1 (amino acid 234–313, UniProt KB: P15941) and MUC2 (amino acid 2008–2168, UniProt KB: Q02817) were cloned into the pGEX-5X-1 vector. GST-tagged proteins were expressed in Rosetta-gami 2(DE3) pLysS strain (Novagen, Germany) and purified by glutathione affinity chromatography using GST affinity resin (Smart-Lifesciences, China).

### Lectin blot

The purified protein substrate was separated by SDS-PAGE and transferred to cellulose acetate membranes (GE Healthcare) at 15 V for 40 min. The membrane was washed with PBS containing 1% Tween 20 (PBST buffer) at room temperature. After that, the membrane was incubated with *Vicia villosa* agglutinin (primarily recognizing Tn antigen of O-GalNAc glycosylation) conjugated with HRP (Vector Laboratories, Burlingame, CA, USA) in PBST for 1 h. The blots were visualized by ECL (Pierce).

### Functional validation of candidate mucinases

#### Enzyme quantity scale

Mucinases (0–50 ng) and substrates (500 ng) were incubated with candidate mucinases at 37°C for 8 h. Digestion products were separated by SDS-PAGE and visualized by silver staining.

#### Time scale

Mucinases (50 ng) and substrates (500 ng) were incubated with candidate mucinases at 37°C for 0–2 h. Digestion products were separated by SDS-PAGE and visualized by silver staining.

### Statistics and data visualization

PCA was performed by the vegan package of R and tested by PERMANOVA with permutation set to 999. Procrustes analysis was conducted in R using Bray-Curtis distance for microbiome data and Euclidean distance. *P* values were obtained from 999 permutation tests. The Logistic Regression, Random Forest, and Support Vector Machine models were constructed and analyzed in R. PLS-DA was performed using in-house scripts, and an unpaired *t*-test was utilized and adjusted by the Benjamini-Hochberg method in MATLAB 2024b. The ggplot2 package was applied for visualization. Multiple sequence alignment images were generated using BioEdit. The protein structure comparison was performed by PyMOL (43), and the phylogenetic tree was constructed by MEGA (42). Some of the diagrams in the flowchart of our study were created with BioRender (https://www.biorender.com/).

## Data Availability

The metagenomic sequencing data we used in this study are publicly available in NCBI SRA with accession no. PRJNA731589. All the computationally screened mucinases in this study were obtained with these metagenomic data.

## SUPPLEMENTAL MATERIAL

Supplemental figures aem.01903-25-s0001.pdf Figures S1 to S4.

ASM does not own the copyrights to Supplemental Material that may be linked to, or accessed through, an article. The authors have granted ASM a non-exclusive, world-wide license to publish the Supplemental Material files. Please contact the corresponding author directly for reuse.

## References

[1] Di Tommaso N, Gasbarrini A, Ponziani FR. 2021. Intestinal barrier in human health and disease. Int J Environ Res Public Health 18:12836. doi:10.3390/ijerph182312836 34886561 PMC8657205

[2] Ahmad Kendong SM, Raja Ali RA, Nawawi KNM, Ahmad HF, Mokhtar NM. 2021. Gut dysbiosis and intestinal barrier dysfunction: potential explanation for early-onset colorectal cancer. Front Cell Infect Microbiol 11:744606. doi:10.3389/fcimb.2021.744606 34966694 PMC8710575

[3] Genua F, Raghunathan V, Jenab M, Gallagher WM, Hughes DJ. 2021. The role of gut barrier dysfunction and microbiome dysbiosis in colorectal cancer development. Front Oncol 11:626349. doi:10.3389/fonc.2021.626349 33937029 PMC8082020

[4] Wu M, Wu Y, Li J, Bao Y, Guo Y, Yang W. 2018. The dynamic changes of gut microbiota in Muc2 deficient mice. Int J Mol Sci 19:2809. doi:10.3390/ijms19092809 30231491 PMC6164417

[5] Inaba R, Vujakovic S, Bergstrom K. 2023. The gut mucus network: a dynamic liaison between microbes and the immune system. Semin Immunol 69:101807. doi:10.1016/j.smim.2023.101807 37478802

[6] Ma J, Piao X, Mahfuz S, Long S, Wang J. 2022. The interaction among gut microbes, the intestinal barrier and short chain fatty acids. Anim Nutr 9:159–174. doi:10.1016/j.aninu.2021.09.012 35573092 PMC9079705

[7] Imai Y, Yamagishi H, Fukuda K, Ono Y, Inoue T, Ueda Y. 2013. Differential mucin phenotypes and their significance in a variation of colorectal carcinoma. World J Gastroenterol 19:3957–3968. doi:10.3748/wjg.v19.i25.3957 23840140 PMC3703182

[8] Chen C, Patel A, Demirkhanyan L, Gondi CS. 2025. The role of mucins in cancer and cancer progression: a comprehensive review. Curr Issues Mol Biol 47:406. doi:10.3390/cimb47060406 40699805 PMC12191488

[9] Grondin JA, Kwon YH, Far PM, Haq S, Khan WI. 2020. Mucins in intestinal mucosal defense and inflammation: learning from clinical and experimental studies. Front Immunol 11:2054. doi:10.3389/fimmu.2020.02054 33013869 PMC7500085

[10] Liu Y, Yu Z, Zhu L, Ma S, Luo Y, Liang H, Liu Q, Chen J, Guli S, Chen X. 2023. Orchestration of MUC2 — The key regulatory target of gut barrier and homeostasis: a review. Int J Biol Macromol 236:123862. doi:10.1016/j.ijbiomac.2023.123862 36870625

[11] Yousefi M, Dehghani S, Nosrati R, Zare H, Evazalipour M, Mosafer J, Tehrani BS, Pasdar A, Mokhtarzadeh A, Ramezani M. 2019. Aptasensors as a new sensing technology developed for the detection of MUC1 mucin: a review. Biosens Bioelectron 130:1–19. doi:10.1016/j.bios.2019.01.015 30716589

[12] Li X, Wubbolts RW, Bleumink-Pluym NMC, van Putten JPM, Strijbis K. 2021. The transmembrane mucin MUC1 facilitates β1-integrin-mediated bacterial invasion. mBio 12. doi:10.1128/mBio.03491-20 PMC8092303 33824202

[13] Shon DJ, Kuo A, Ferracane MJ, Malaker SA. 2021. Classification, structural biology, and applications of mucin domain-targeting proteases. Biochem J 478:1585–1603. doi:10.1042/BCJ20200607 33909028

[14] Taleb V, Liao Q, Narimatsu Y, García-García A, Compañón I, Borges RJ, González-Ramírez AM, Corzana F, Clausen H, Rovira C, Hurtado-Guerrero R. 2022. Structural and mechanistic insights into the cleavage of clustered O-glycan patches-containing glycoproteins by mucinases of the human gut. Nat Commun 13:4324. doi:10.1038/s41467-022-32021-9 35882872 PMC9325726

[15] Paone P, Cani PD. 2020. Mucus barrier, mucins and gut microbiota: the expected slimy partners? Gut 69:2232–2243. doi:10.1136/gutjnl-2020-322260 32917747 PMC7677487

[16] Raba G, Luis AS. 2023. Mucin utilization by gut microbiota: recent advances on characterization of key enzymes. Essays Biochem 67:345–353. doi:10.1042/EBC20220121 36695502 PMC10154618

[17] Li Y, Cao H. 2022. Gut microbiota signatures in tumor, para-cancerous, normal mucosa, and feces in colorectal cancer patients. Front Cell Dev Biol 10. doi:10.3389/fcell.2022.916961 PMC9201480 35721506

[18] Cheng Y, Ling Z, Li L. 2020. The intestinal microbiota and colorectal cancer. Front Immunol 11:615056. doi:10.3389/fimmu.2020.615056 33329610 PMC7734048

[19] Wong CC, Yu J. 2023. Gut microbiota in colorectal cancer development and therapy. Nat Rev Clin Oncol 20:429–452. doi:10.1038/s41571-023-00766-x 37169888

[20] Wang T, Cai G, Qiu Y, Fei N, Zhang M, Pang X, Jia W, Cai S, Zhao L. 2012. Structural segregation of gut microbiota between colorectal cancer patients and healthy volunteers. ISME J 6:320–329. doi:10.1038/ismej.2011.109 21850056 PMC3260502

[21] Tan B, Qiu Y, Zou X, Chen T, Xie G, Cheng Y, Dong T, Zhao L, Feng B, Hu X, Xu LX, Zhao A, Zhang M, Cai G, Cai S, Zhou Z, Zheng M, Zhang Y, Jia W. 2013. Metabonomics identifies serum metabolite markers of colorectal cancer. J Proteome Res 12:3000–3009. doi:10.1021/pr400337b 23675754 PMC5902797

[22] Shon DJ, Malaker SA, Pedram K, Yang E, Krishnan V, Dorigo O, Bertozzi CR. 2020. An enzymatic toolkit for selective proteolysis, detection, and visualization of mucin-domain glycoproteins. Proc Natl Acad Sci USA 117:21299–21307. doi:10.1073/pnas.2012196117 32817557 PMC7474620

[23] Noach I, Ficko-Blean E, Pluvinage B, Stuart C, Jenkins ML, Brochu D, Buenbrazo N, Wakarchuk W, Burke JE, Gilbert M, Boraston AB. 2017. Recognition of protein-linked glycans as a determinant of peptidase activity. Proc Natl Acad Sci USA 114:E679–E688. doi:10.1073/pnas.1615141114 28096352 PMC5293097

[24] Bakshani CR, Ojuri TO, Pilgaard B, Holck J, McInnes R, Kozak RP, Zakhour M, Çakaj S, Kerouedan M, Newton E, Bolam DN, Crouch LI. 2025. Carbohydrate-active enzymes from Akkermansia muciniphila break down mucin O-glycans to completion. Nat Microbiol 10:585–598. doi:10.1038/s41564-024-01911-7 39891011 PMC11790493

[25] Sheng Q-S, He K-X, Li J-J, Zhong Z-F, Wang F-X, Pan L-L, Lin J-J. 2020. Comparison of gut microbiome in human colorectal cancer in paired tumor and adjacent normal tissues. Onco Targets Ther 13:635–646. doi:10.2147/OTT.S218004 32021305 PMC6982458

[26] Ioannou A, Berkhout MD, Geerlings SY, Belzer C. 2025. Akkermansia muciniphila: biology, microbial ecology, host interactions and therapeutic potential. Nat Rev Microbiol 23:162–177. doi:10.1038/s41579-024-01106-1 39406893

[27] Cani PD, Depommier C, Derrien M, Everard A, de Vos WM. 2022. Akkermansia muciniphila: paradigm for next-generation beneficial microorganisms. Nat Rev Gastroenterol Hepatol 19:625–637. doi:10.1038/s41575-022-00631-9 35641786

[28] Abramson J, Adler J, Dunger J, Evans R, Green T, Pritzel A, Ronneberger O, Willmore L, Ballard AJ, Bambrick J, et al.. 2024. Accurate structure prediction of biomolecular interactions with AlphaFold 3. Nature 630:493–500. doi:10.1038/s41586-024-07487-w 38718835 PMC11168924

[29] Muhammed MT, Aki-Yalcin E. 2024. Molecular docking: principles, advances, and its applications in drug discovery. LDDD 21:480–495. doi:10.2174/1570180819666220922103109

[30] Fu LM, Niu BF, Zhu ZW, Wu ST, Li WZ. 2012. CD-HIT: accelerated for clustering the next-generation sequencing data. Bioinformatics 28:3150–3152. doi:10.1093/bioinformatics/bts565 23060610 PMC3516142

[31] Potter SC, Luciani A, Eddy SR, Park Y, Lopez R, Finn RD. 2018. HMMER web server: 2018 update. Nucleic Acids Res 46:W200–W204. doi:10.1093/nar/gky448 29905871 PMC6030962

[32] Chen SF, Zhou YQ, Chen YR, Gu J. 2018. Fastp: an ultra-fast all-in-one FASTQ preprocessor. Bioinformatics 34:i884–i890. doi:10.1093/bioinformatics/bty560 30423086 PMC6129281

[33] Langmead B, Salzberg SL. 2012. Fast gapped-read alignment with Bowtie 2. Nat Methods 9:357–359. doi:10.1038/nmeth.1923 22388286 PMC3322381

[34] Li DH, Luo RB, Liu CM, Leung CM, Ting HF, Sadakane K, Yamashita H, Lam TW. 2016. MEGAHIT v1.0: a fast and scalable metagenome assembler driven by advanced methodologies and community practices. Methods 102:3–11. doi:10.1016/j.ymeth.2016.02.020 27012178

[35] Danecek P, Bonfield JK, Liddle J, Marshall J, Ohan V, Pollard MO, Whitwham A, Keane T, McCarthy SA, Davies RM, Li H. 2021. Twelve years of SAMtools and BCFtools. Gigascience 10:giab008. doi:10.1093/gigascience/giab008 33590861 PMC7931819

[36] Kang DD, Li F, Kirton E, Thomas A, Egan R, An H, Wang Z. 2019. MetaBAT 2: an adaptive binning algorithm for robust and efficient genome reconstruction from metagenome assemblies. Peerj 7:e7359. doi:10.7717/peerj.7359 31388474 PMC6662567

[37] Chklovski A, Parks DH, Woodcroft BJ, Tyson GW. 2023. CheckM2: a rapid, scalable and accurate tool for assessing microbial genome quality using machine learning. Nat Methods 21:735–735. doi:10.1038/s41592-023-01940-w 38514780

[38] Olm MR, Brown CT, Brooks B, Banfield JF. 2017. dRep: a tool for fast and accurate genomic comparisons that enables improved genome recovery from metagenomes through de-replication. ISME J 11:2864–2868. doi:10.1038/ismej.2017.126 28742071 PMC5702732

[39] Chaumeil PA, Mussig AJ, Hugenholtz P, Parks DH. 2022. GTDB-Tk v2: memory friendly classification with the genome taxonomy database. Bioinformatics 38:5315–5316. doi:10.1093/bioinformatics/btac672 36218463 PMC9710552

[40] Aroney STN, Newell RJP, Nissen JN, Camargo AP, Tyson GW, Woodcroft BJ. 2025. CoverM: read alignment statistics for metagenomics. Bioinformatics 41:btaf147. doi:10.1093/bioinformatics/btaf147 40193404 PMC11993303

[41] Li H, Wu G, Zhao L, Zhang M. 2021. Suppressed inflammation in obese children induced by a high-fiber diet is associated with the attenuation of gut microbial virulence factor genes. Virulence 12:1754–1770. doi:10.1080/21505594.2021.1948252 34233588 PMC8274444

[42] Tamura K, Dudley J, Nei M, Kumar S. 2007. MEGA4: molecular evolutionary genetics analysis (MEGA) software version 4.0. Mol Biol Evol 24:1596–1599. doi:10.1093/molbev/msm092 17488738

[43] Yuan S, Chan HCS, Hu Z. 2017. Using PyMOL as a platform for computational drug design. WIREs Comput Mol Sci 7:e1298. doi:10.1002/wcms.1298

