# Supplementary material for: Enhanced microbiota-derived mucinases in colorectal cancer patients revealed by gut metagenome probing coupled with functional validation
Source: Appl Environ Microbiol. 2026 Apr 6;92(5):e01903-25. doi: 10.1128/aem.01903-25 (PMC13188911; doi:10.1128/aem.01903-25)
Supplement: Supplemental figures — Figures S1 to S4. [file aem.01903-25-s0001.pdf]

Supplementary material

Systematiclly enhanced microbiota-derived mucinases in colorectal cancer patients revealed by gut metagenomic modeling and functional validation

Including:

Supplementary Figures 1-4

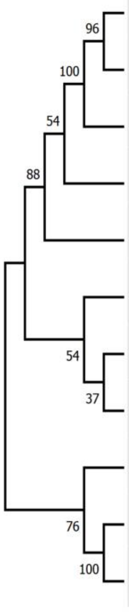

Mucinase	Organism	MEROPS
BT4244	Bacteroides thetaiotaomicron	M60
Amuc0908	Akkermansia muciniphila	M60
Amuc0627	Akkermansia muciniphila	M60
Amuc1514	Akkermansia muciniphila	M98
IMPa	Pseudomonas aeruginosa	M88
Pic	Escherichia coli	S6
OgpA	Akkermansia muciniphila	M11
StcE	Escherichia coli	M66
CpaA	Acinetobacter	M72
ZmpC	Streptococcus pneumoniae	M26
ZmpB	Clostridium perfringens	M60

Supplementary Fig 1 The bacterial sources and functional family classification of the 11 mucinases. This figure has been compiled based on the article by D. Judy Shon et al.

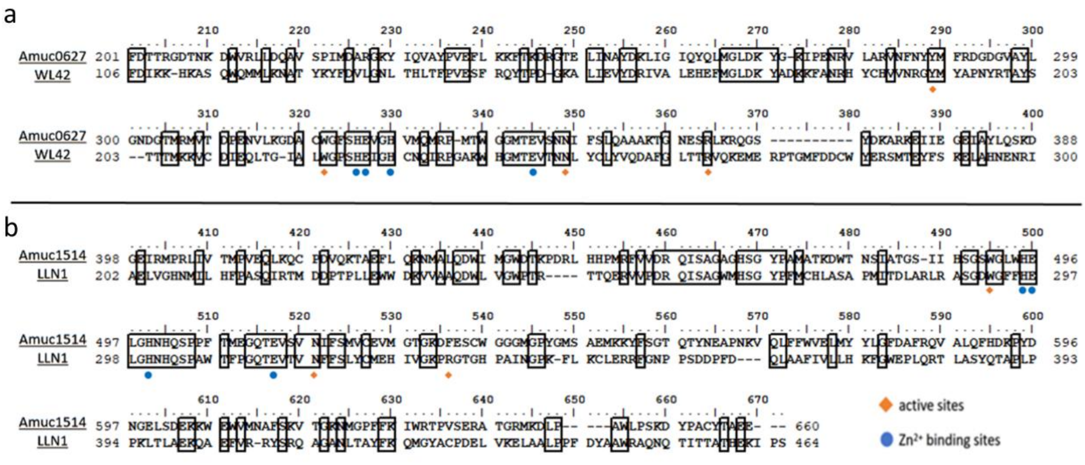

Supplementary Fig 2 The sequence comparison of (a) Amuc0627 and WL42 and comparison of (b) Amuc1514 and LLN1 proteins.The active sites and the Zn^{2+} ion binding sites are respectively marked with orange rhombuses and blue circles.

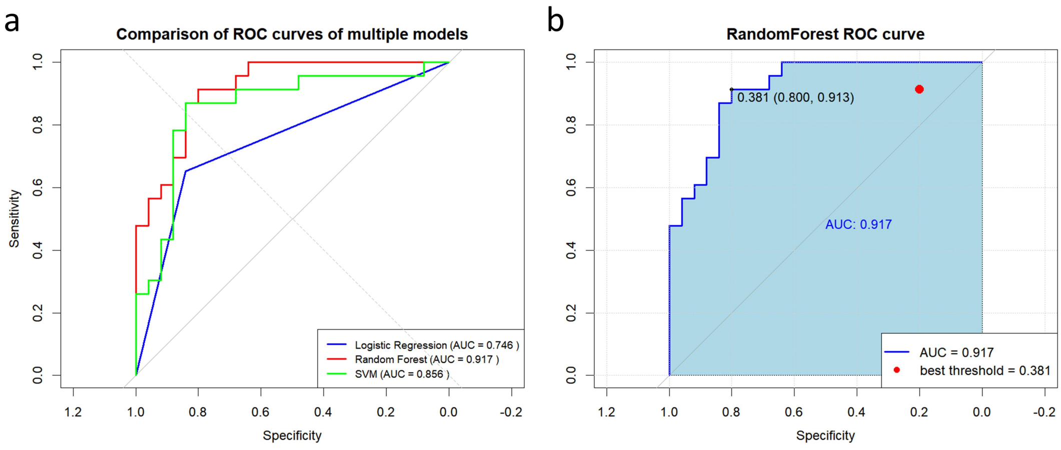

Supplementary Fig 3 The machine learning prediction models constructed using 42 mucinases. (a) Comparison of ROC curves for the three models: Logistic Regression, Random Forest, and SVM. (b) The ROC curve of the Random Forest model, and the calculation of its AUC and the best threshold.

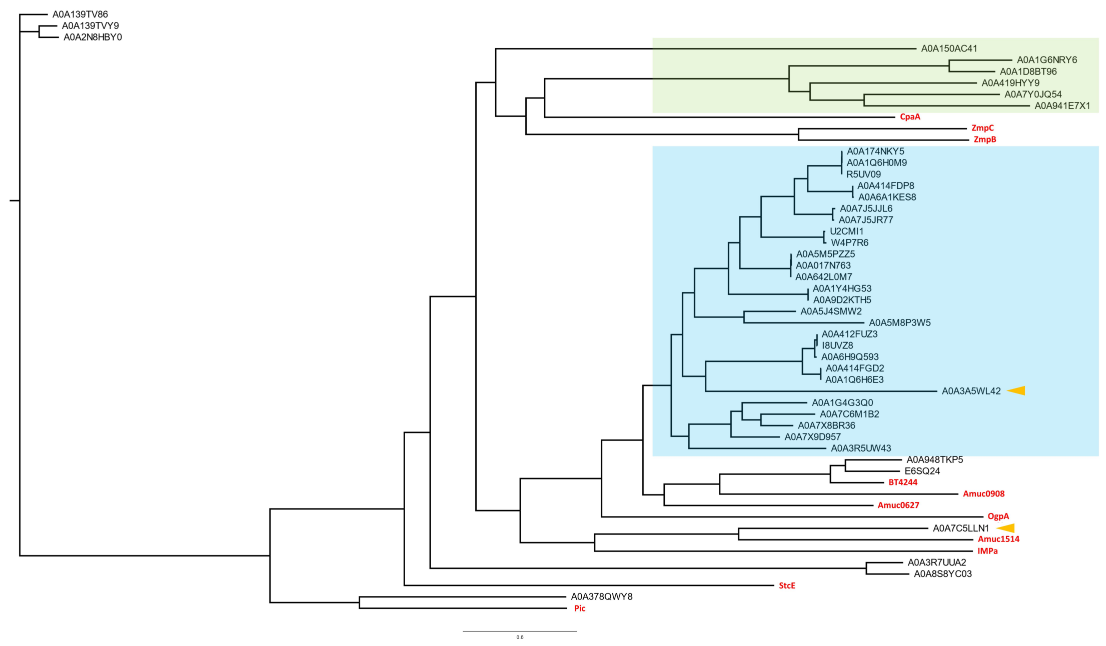

Supplementary Fig 4 The phylogenetic tree of 42 candidate mucinases and 11 initial mucinases. The names of 11 known mucinases are marked in red, and the orange triangles indicate the two selected mucinases. The blue and green areas respectively show the two main clusters of candidate mucinases.
